# Depth-associated selection and drift shape persistent microbial populations in Holocene lake sediments

**DOI:** 10.1128/msystems.01500-25

**Published:** 2026-05-05

**Authors:** Paula Rodríguez, Sophie A. Simon, Alexander J. Probst, Cara Magnabosco

**Affiliations:** 1 Department of Earth and Planetary Sciences, ETH Zurich 111841 , Zurich, Switzerland; 2 Environmental Metagenomics, Faculty of Chemistry, Research Center One Health Ruhr of the University Alliance Ruhr, University of Duisburg-Essen, Essen, Germany; 3 Centre of Water and Environmental Research (ZWU), University of Duisburg-Essen, Essen, Germany; 4 Department of Energy Joint Genome Institute, Lawrence Berkeley National Laboratory, Berkeley, California, USA; University of Hawaii at Manoa, Kaneohe, Hawaii, USA

**Keywords:** lacustrine sediments, pangenomics

## Abstract

Cosmopolitan microbial lineages are found in anoxic sediments worldwide, but the details about their ecology and evolution remain underexplored. In this study, we identified persistent populations from these cosmopolitan lineages belonging to *Planctomycetes*, *Chloroflexi Atribacteria*, and *Candidatus* Bathyarchaeia from an ~8,000-year sedimentary sequence. To investigate the genomic variations within these persistent populations, a pangenome of each population was constructed using all non-redundant metagenome-assembled genomes (MAGs) recovered from the sedimentary sequence and was screened for enriched functional genes, single-nucleotide polymorphism (SNP) density, *dN*/*DS* ratios, and pseudogene content. Our results show that the majority of persistent populations studied possess large variable genomes enriched for energy conservation and transcriptional regulation functions with increasing depth, whereas *Planctomycetes* retain a highly conserved, SNP-poor core genome. Analysis of SNPs across depths indicates progressive isolation with burial, while a subset of core genes shows signatures of positive selection. Collectively, the data support depth-associated selection acting alongside drift across Lake Cadagno’s persistent sedimentary lineages.

**IMPORTANCE:**

Throughout the subsurface, multiple examples of “evolutionary stasis” have been reported in microbial lineages that exhibit lower rates of metabolic activity and cellular turnover. This study uses an ~8,000-year sedimentary record of Lake Cadagno to evaluate how persistent populations of cosmopolitan bacteria and archaea have changed with burial and identifies signals of progressive genetic isolation along with positive selection of population-specific subsets of core genes with depth. Together, these changes show that Lake Cadagno’s persistent populations are not in stasis but diverge over time and burial.

## INTRODUCTION

All living organisms depend on energy sourced from the surrounding environment (1). In the surface biosphere, a variety of sources provide a constant flux of electron acceptors and donors to support cellular growth, high metabolic rates, and large population sizes. By contrast, microorganisms that live within the deep sedimentary biosphere are cut off from renewed energy inputs, which is hypothesized to shift cellular activity toward maintenance and survival rather than growth (2). In fact, amino acid racemization models have estimated that deep sedimentary cellular turnover can be one to two orders of magnitude slower than at the surface (3, 4). This decrease in cellular turnover frequently co-occurs with decreases in cell numbers due to cell death as sediment ages increase and the availability of organic carbon decreases (5). Such depth-specific changes have been used to explain global anoxic sediment microbial diversity patterns, such as decreases in species richness and vertical stratification of microbial groups with depth (5).

Previous studies in energy-isolated marine anoxic sediments suggest that “persistent populations,” defined as microbial populations present throughout a sedimentary sequence but becoming dominant at depth, have low mutation rates (6). These persistent populations concentrate within a few taxa, e.g., *Ca*. Bathyarchaeia, *Chloroflexi*, *Planctomycetes*, and *Atribacteria* are globally distributed within anoxic sedimentary ecosystems and, in many cases, have been linked to the active cycling of major elements in these sedimentary environments (e.g., 6–12). The prevalence and activity of these lineages within deep sedimentary environments suggest that these lineages intrinsically possess genomic traits that are needed to survive energy-limited conditions in non-advective sediments and are, therefore, preferentially selected from sedimentary communities during burial (6) while less adapted lineages are lost to extinction at depth. This purifying selection contrasts with an adaptive evolutionary process in which surface- or shallow subsurface-dwelling microorganisms acquire new traits to survive in sedimentary environments during burial.

Recently, studies in anoxic mangrove and cold seep sedimentary environments have demonstrated the diversification of genetic variants in key genes for metabolism within microbial populations (13, 14). These results contrast with traditional models of population-level changes with burial and motivate a reassessment of how microbial populations in non-advective sediments change with burial. To address this, we contribute a new example of persistent population microdiversity patterns from anoxic sediments isolated from fresh organic carbon and surface inputs for thousands of years in Lake Cadagno, Switzerland. Lake Cadagno provides a well-constrained study site, with a suitable sedimentary record that reflects the redox changes in the water column since the formation of the lake in the last glacial and a lithostratigraphic profile that contains sediments with organic carbon from lacustrine and terrestrial sources (10, 15–17). By using pangenomics to evaluate the intra-population genomic variability of these persistent populations across sediment depths, we provide new insights into the eco-evolutionary processes driving community assembly in lacustrine anoxic sediments, showing distinct examples of adaptive evolution, purifying selection, and genetic drift within distinct populations of the sedimentary community.

## RESULTS

### Persistent bacterial and archaeal populations in Lake Cadagno

**Fig 1:**
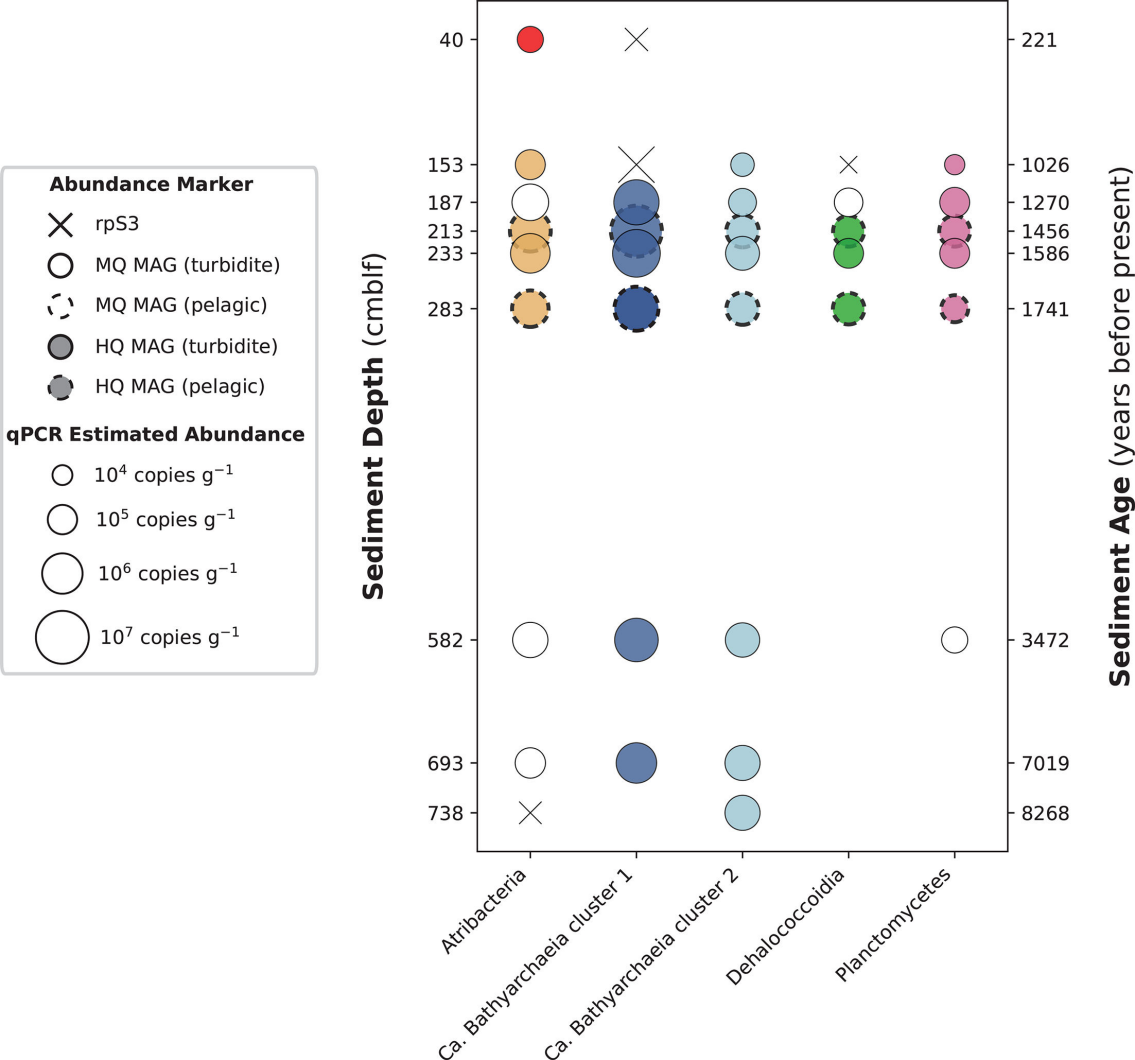
The depth-specific quantitative PCR (qPCR) estimated abundances (marker size) of Lake Cadagno persistent populations (*x*-axis) are shown. Medium quality (MQ) MAGs (open circles) and ribosomal protein S3 nucleotide sequences (rpS3, crosses) with >95% ANI to high quality (HQ) MAGs are added for completeness. Marker borders indicate whether the MAG was derived from a pelagic (dotted border) or turbidite (smooth border) sediment sample. The *Atribacteria* MAG at 40 cm depth (red circle) is a 97.5% ANI relative of the *Atribacteria* persistent population (gold circles) and, therefore, was excluded from our pangenomic study. Additional information on the relative, normalized, and 16S rRNA gene-derived abundance of persistent population clusters with depth can be found in Fig. S1.

A total of five persistent population genome clusters with >98.5% average nucleotide identity (ANI) and represented by high quality metagenome-assembled genomes (MAGs; CheckM [18], >90% completeness, <5% contamination) were recovered from at least three sediment depths ranging from 153 to 738 cm below lake floor (cmblf) with sediment ages of 1,026–8,268 years before present in the Lake Cadagno data set (Fig. 1; Table S1). Among these, two persistent archaeal clusters affiliated with *Ca*. Bathyarchaeia were identified, whereas each of the persistent bacterial taxonomic groups (*Atribacteria*, *Dehalococcoidia* of the phylum *Chloroflexi*, and *Planctomycetes*) was represented by a single persistent cluster (Table 1 and Fig. 1; Table S1). All high-quality persistent MAGs range in size from 1.35 ± 0.18 × 10^6^ nucleotides (nt) to 3.86 ± 0.02 × 10^6^ nt, with protein-coding densities of 85%–89% (Table 1). Throughout the sediment sequence, the persistent *Planctomycetes* population cluster exhibits the lowest relative and absolute abundances, while *Ca*. Bathyarchaeia cluster 1 (BC1) is the most abundant persistent group throughout the sedimentary sequence (Fig. 1; Fig. S1). With the exception of the *Ca*. Bathyarchaeia cluster 2 (BC2) population, which becomes more abundant at depths >600 cmblf, all population clusters are most abundant in absolute and relative terms in the ~200–300 cmblf organic carbon remineralization zone that exhibits locally elevated concentrations of phosphate, ammonium, iron, and manganese (10, 15) in the sedimentary sequence (Fig. 1; Fig. S1).

**TABLE 1:** Average genome statistics for persistent population clusters at 98% ANI^*a*^

Lake Cadagno persistent population cluster	Metagenome assembled genome metrics					CheckM2 metrics (%)		GTDB
	Size (Mb)	No. of contigs	N50 (kb)	% GC	PCD (%)	Completeness	Contamination	Relatives
*Ca*. Bathyarchaeia cluster 1 (*n* = 6)	1.35 ± 0.18	36.3 ± 10.3	76 ± 42	48.1 ± 0.2	87	96.2 ± 2.7	4.2 ± 0.7	1^⋆^
*Ca*. Bathyarchaeia cluster 2 (*n* = 8)	2.04 ± 0.20	61.1 ± 24.7	76 ± 41	56.8 ± 0.7	87	94.7 ± 1.7	1.8 ± 0.6	0
*Atribacteria* (*n* = 4)	1.80 ± 0.07	77.8 ± 23.1	48 ± 17	33.1 ± 0.1	85	97.4 ± 1.5	0.6 ± 1.1	7^⊘^
*Planctomycetes* (*n* = 5)	3.86 ± 0.02	79.4 ± 65.0	292 ± 346	67.7 ± 0.0	89	98.1 ± 0.9	0.9 ± 0.5	0
*Dehalococcoidia* (*n* = 3)	1.46 ± 0.47	27.0 ± 16.1	151 ± 66	46.1 ± 0.1	88	93.5 ± 1.8	1.7 ± 2.3	2^†^

^*a*^ The values represent the mean genome statistics calculated across all MAGs within each cluster, where *n* is the number of high-quality genomes identified in the cluster. Additional information on these MAGs is included in Table S1. Mb = 106 nucleotides. kb = 103 nucleotides. PCD = protein coding density. Genome Taxonomy Database (GTDB) Species Representative of GTDB Relatives: ^⋆^GCA_022865455.1; ^⊘^GCA_002782675.1; ^†^GCA_001872925.1.

Annotation of the persistent bacterial population clusters assigns the *Atribacteria*, *Dehalococcoidia*, and *Planctomycetes* clusters to the JS1 lineage, GIF9 clade, and the candidatus genus *Ca*. Brocadia, respectively. BC1 belongs to the order “Baizomonadales” (19) and BC2 is a member of the order “Wuzhiqibiales” (19). Species-level relatives (Genome Taxonomy Database, GTDB, R09-RS220 [20]) for BC1 and the *Atribacteria* and *Dehalococcoidia* persistent populations were identified in metagenomes from the Siberian “Polar Fox Lagoon” sediments (BC1) and Utah, USA’s “Crystal Geyser” (*Atribacteria* and *Dehalococcoidia*) (Table 1). The GTDB representative MAGs for these taxa exhibited 95.0% ± 1.5%, 97.0% ± 1.8%, and 98.0% ± 0.6% ANI with the persistent Lake Cadagno *Atribacteria*, *Dehalococcoidia*, and BC1 MAGs, respectively.

**Fig 2:**
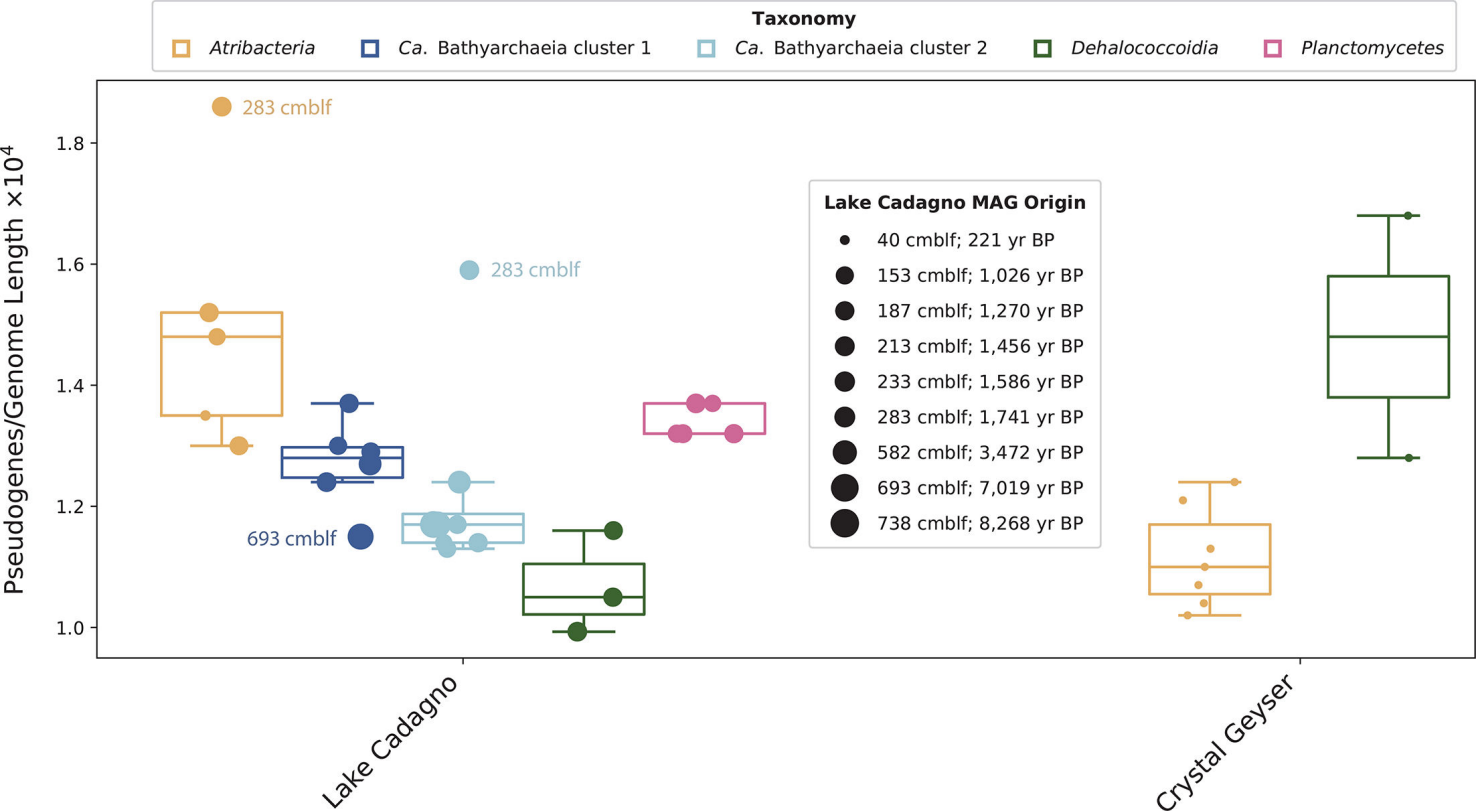
A box plot of the pseudogene count relative to the total genome size (ps/L, *y*-axis) for individual MAGs within a persistent population (*x*-axis) is shown. MAGs whose pseudogene frequencies significantly differ from the rest of the pangenome are labeled with the sediment depth from which they were recovered. For comparison, the pseudogene frequencies of the GTDB relatives of Lake Cadagno Persistent Populations from Crystal Geyser (Table 1) are also shown, and the pseudogene frequency of the Polar Fox Lagoon *Ca*. Bathyarchaeia Cluster 1 MAG (GCA_022865455.1) is 1.28 × 10^−^4.

Within each high-quality MAG, between 125 and 527 pseudogenes (fragments of, presumably, once-functional genes that have been silenced by one or more deleterious mutations) were identified with the largest MAGs (e.g., *Planctomycetes*) containing the most pseudogenes (Table S2). To control for the scaling of pseudogenes with genome size, we assessed pseudogene frequencies with respect to genome length (ps/L). Using this metric, *Atribacteria* exhibit the highest ps/L and are the only population cluster to show an increase in ps/L with depth (Fig. 2; Table S2). Interestingly, 70% of Lake Cadagno’s *Atribacteria* pseudogenes are uniquely encountered in the deepest high-quality MAG (283 cmblf) and relate to multiple COG categories (e.g., amino acid transport and metabolism [E], energy production and conversion [C], replication, recombination, and repair [L], and inorganic ion transport and metabolism [P]) (Table S2). By contrast, BC1 is the only population cluster to exhibit a decrease in ps/L with depth (Fig. 2; Table S2) and the deepest BC1 high-quality MAG is also the smallest genome (1.1 Mb) within the population cluster (Table S1).

### Microdiversity of persistent population clusters

**Fig 3:**
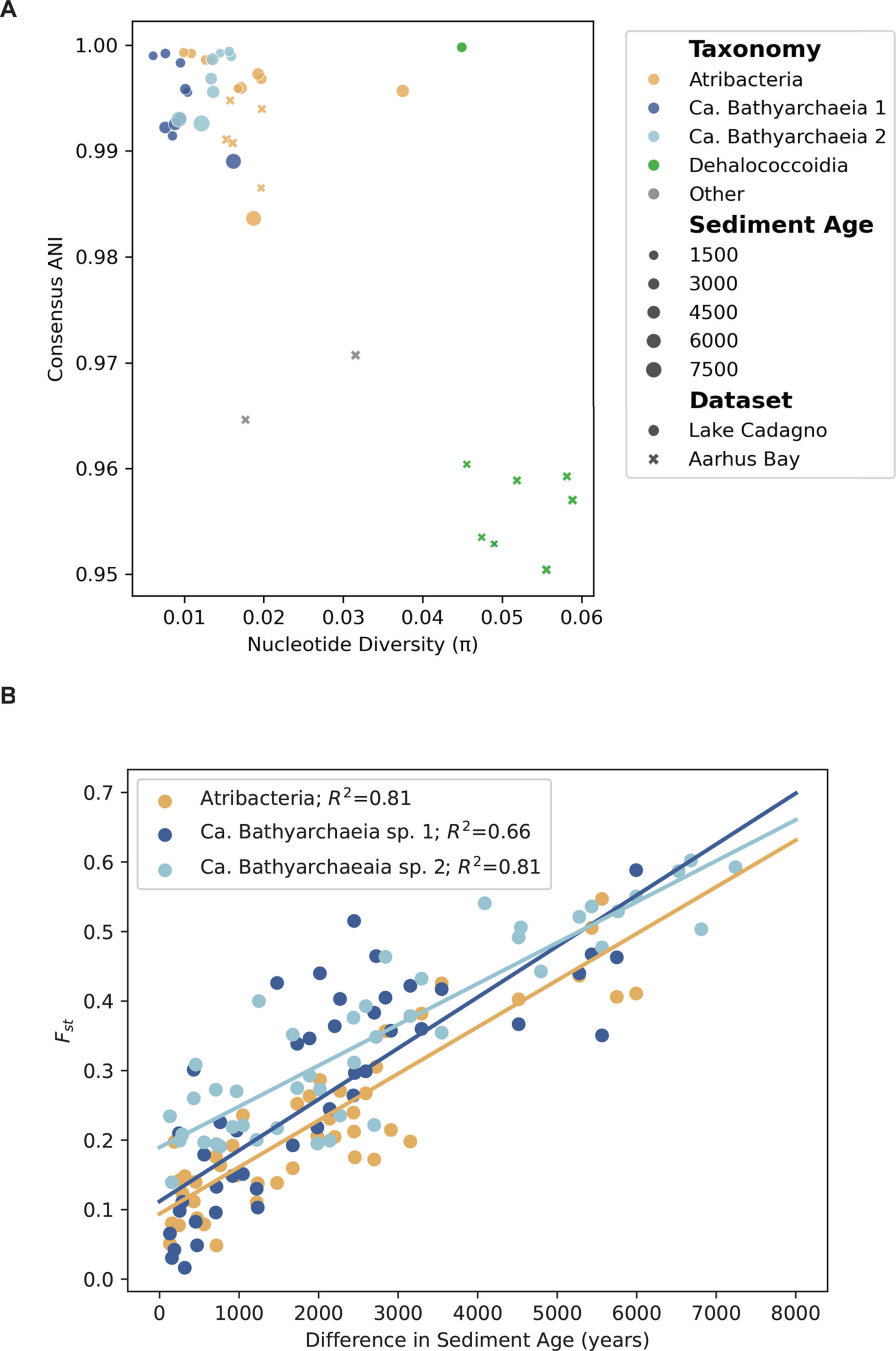
(**A**) inStrain-computed consensus ANI versus rarefied nucleotide diversity (π) for persistent populations from Lake Cadagno (circles) and Aarhus Bay (×). Colors denote major taxa and point sizes reflect sediment age (years). It is worth noting that the sediment age of the Aarhus Bay MAGs included in this analysis is derived from sediments deposited from approximately 250 to 1,750 years before present. (**B**) The pairwise fixation index (*F*_ST_) of Lake Cadagno MAGs as a function of the absolute difference in sediment age between samples is shown with an ordinary-least-squares fit. *R*^2^ values for the fits are indicated in the legend.

To assess the intra-population diversity of Lake Cadagno and compare it to an earlier microdiversity study of a similarly aged (5,000 year) anoxic sedimentary sequence from Aarhus Bay (6), we mapped metagenomic reads from each Lake Cadagno or Aarhus Bay sample to the representative genome of each population cluster and calculated various microdiversity metrics using BWA (21) and inStrain (22), respectively (see Materials and Methods for further details). This analysis reveals that the Lake Cadagno intra-population nucleotide diversity (π), consensus ANI, and recombination are lower than what is observed within persistent populations of Aarhus Bay (Fig. 3A; Fig. S2). Despite the low diversity of each persistent population, we observed progressive divergence in population allele frequencies across Lake Cadagno’s 8,000-year sedimentary sequence, as reflected by an approximately linear increase in the pairwise fixation index (*F*_ST_) with the differences in sediment age (Fig. 3B). *F*_ST_, a measure of genetic differentiation among populations as defined by reference 23, increases from around 0 to ~0.6 as differences in sediment age increase to values beyond 7,000 years. The lesser sequencing depth and greater divergence of populations with depth in Aarhus Bay’s 5,000-year sedimentary sequence prohibit a direct comparison of *F*_ST_ versus sediment age patterns; however, given the lower consensus ANI between depth-separated populations, we should expect even higher *F*_ST_ values in Aarhus Bay.

### Pangenomes of persistent populations

**Fig 4:**
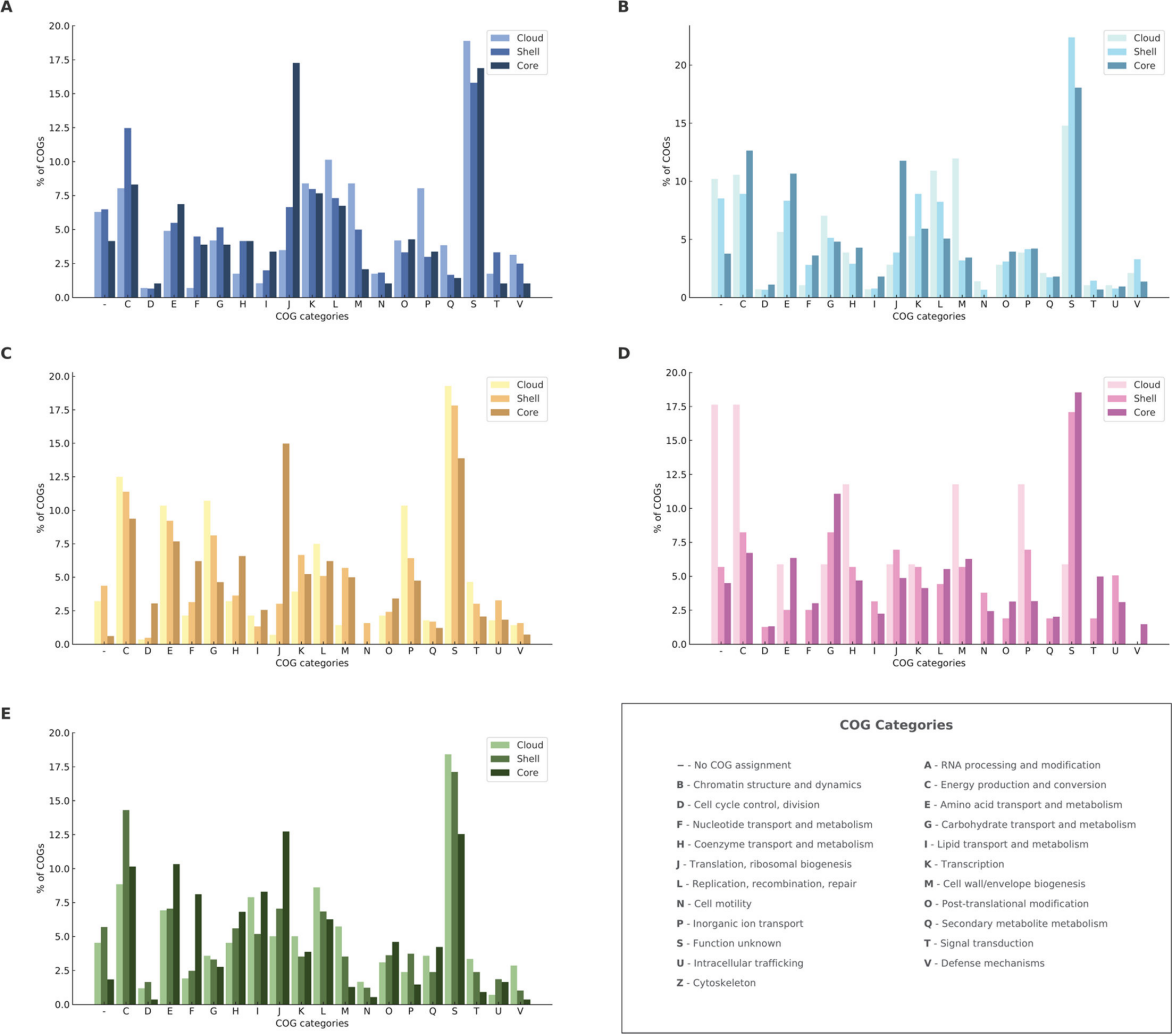
Distribution of COG functional categories across core, shell, and cloud genome partitions for persistent population clusters. Bars represent the percentage of COGs assigned to each functional category within each partition. (**A**) *Ca*. Bathyarchaeia cluster 1 local pangenome; (**B**) *Ca*. Bathyarchaeia cluster 2 local pangenome; (**C**) *Atribacteria* local pangenome; (**D**) *Planctomycetes* local pangenome; (**E**) *Dehalococcoida* global pangenome containing three MAGs from Lake Cadagno (this study) and two MAGs from Crystal Geyser (USA).

To better assess depth-specific differences in genome content with depth, Lake Cadagno-specific (local) pangenomes were constructed for the persistent population clusters (Table 2). Global pangeomes were additionally made for BC1, *Atribacteria*, and *Dehalococcoidia* by including related MAGs from non-Lake Cadagno sample origins for comparison (Table 3). Both persistent *Ca*. Bathyarchaeia clusters (BC1, BC2) and the persistent *Atribacteria* population cluster exhibit fairly large variable genomes (60%–70% of the local pangenome; Table 2), whereas the core genome of *Planctomycetes* accounts for 90% of the group’s local pangenome. Across all pangenomes, core genomes are predominantly represented by COG categories J (translation and ribosomal biogenesis) and S (function unknown), while the variable genomes of the pangenomes all exhibit a greater proportion of replication, recombination, and repair genes (COG category L) relative to the core genome (Fig. 4). No integrated viral sequences were identified in any of the persistent MAGs.

**TABLE 2:** Pangenome statistics for persistent Lake Cadagno population clusters^*a*^

Local pangenome	Total genes	Core genes	Shell genes	Cloud genes	+Sel./Core(%)	SNPs/100 kb
*Ca*. Bathyarchaeia cluster 1 (*n* = 6)	2,291	872 (38.1%)	874 (38.1%)	545 (23.8%)	79/872 (9.1%)	2,193
*Ca*. Bathyarchaeia cluster 2 (*n* = 8)	3,250	1,210 (37.2%)	1,468 (45.2%)	572 (17.6%)	19/1,210 (1.6%)	1,144
*Atribacteria* (*n* = 5)	2,265^*b*^	740 (32.7%)^*b*^	1,073 (47.4%)^*b*^	452 (20.0%)^*b*^	59/740 (8.0%)^*b*^	2,144^*b*^
*Planctomycetes* (*n* = 5)	3,209	2,897 (90.3%)	226 (7.0%)	86 (2.7%)	81/2,940 (2.7%)	30
*Dehalococcoidia* (*n* = 3)	1,691^*c*^	1,200 (71.0%)^*c*^	187 (11.1%)^*c*^	304 (18.0%)^*c*^	NA	8^*c*^

^*a*^ The core genome consists of genes present in 100% of MAGs within a cluster. The shell genome comprises gene families present in any integer number n of MAGs between 2 and *N* − 1 (inclusive), where *N* is the total number of MAGs in the cluster. The cloud genome contains genes found in only a single MAG. The number of core genes under positive selection (+Sel., defined by *dN*/*dS* > 1) was estimated using codeml. SNPs = single-nucleotide polymorphisms of the core genome.

^*b*^ A high-quality *Atribacteria* MAG with 97.5% ANI to the persistent *Atribacteria* cluster from 40 cm below Lake Cadagno’s lake floor was included in this calculation.

^*c*^ No Crystal Geyser *Dehalococcoida* MAGs were included in this calculation.

**TABLE 3:** Pangeome statistics for persistent Lake Cadagno population clusters and their GTDB relatives^*a*^

Global pangenome	Total genes	Core genes	Shell genes	Cloud genes	SNPs/100 kb	Global MAG origin
BC1 (*n* = 7)	2,864	851 (29.7%)	1,023 (35.7%)	990 (34.6%)	2,382	Polar Fox Lagoon, RU
*Atribacteria* (*n* = 12)	3,102	461 (14.9%)	1,991 (64.2%)	650 (21.0%)	4,580	Crystal Geyser, USA
*Dehalococcoidia* (*n* = 5)	2,198	539 (24.5%)	1,084 (49.3%)	575 (26.2%)	2,795	Crystal Geyser, USA

^*a*^ Additional details regarding the non-Lake Cadagno MAGs included in the global pangenomes can be found in Table 1. BC1 = *Ca*. Bathyarchaeia cluster 1. RU = Russia. USA = United States of America.

An evaluation of variable gene content with respect to depth shows significant depth-related changes within the BC2 and *Atribacteria* local pangenomes (Table S3). Within their respective groups, the deepest MAGs share the most genes: BC2 at 693 and 738 cmblf share 106 genes with one another, and *Atribacteria* at 213, 233, and 283 cmblf share 156 genes with one another (Data S1). The shared genes within these populations and at these depths are mostly related to COG categories C (energy production and conservation), E (amino acid transport and metabolism), G (carbohydrate transport and metabolism), J (translation, ribosomal biogenesis), and S (function unknown). Importantly, these differences are not due to long, localized insertions or individual contigs but rather differences in gene content and diversification of protein-encoding genes (Data S1).

Although the *Planctomycetes* local pangenome shares >90% of its genes across depths, 71 KEGG functional ortholog groups (KOs) are significantly enriched in the variable genome, but only one KO is significantly enriched in the core (K12132: protein kinase, *Z*-score: 3.7, Benjamini-Hochberg corrected [BH] *P *value: 0.04, Table S3). Many of the significantly enriched *Planctomycetes* variable genome KOs are related to transport (*n* = 11) and the biosynthesis of cofactors and secondary metabolites (*n* = 14), with the remaining variable genome KOs belonging to a variety of BRITE pathways. *Ca*. Bathyarchaeia variable genomes, on the other hand, are enriched in protein families related to energy conservation and transcriptional regulation (Table S3) but only two BC1 KOs (K03388: HdrA2, *Z*-score: −5.9, BH *P-*value: 2.83 × 10^−6^; K07669: response regulator MprA, Z-score: −4.6, BH *P*-value: 0.003) and one BC2 KO (K03892: ArsR family transcriptional regulator, *Z*-score: −4.3, BH *P*-value: 0.007) pass our significance test.

Nonsynonymous (*dN*) and synonymous (*dS*) substitution rates within each local pangenome’s core genome were additionally calculated using the phylogenetically-aware maximum likelihood algorithm, codeml (24). This analysis identified 238 core genes under positive selection across our local pangenome data set (238/5,762 = 4%; Table 2; Table S4). BC1 and BC2 exhibited the highest (9.1%) and lowest (1.6%) frequencies of core genes under positive selection, respectively. Although over half of the genes under positive selection are poorly classified (including multiple genes assigned to “Function Unknown” COG category S), COG category J (translation and ribosomal biogenesis) is the most common assignment for genes experiencing positive selection in BC1 (*n* = 10) and *Atribacteria* (*n* = 8). COG category G (carbohydrate transport and metabolism) and COG category F (nucleotide transport and metabolism) are the most common assignments for genes experiencing positive selection in *Planctomycetes* (*n* = 11) and BC2 (*n* = 3), respectively (Table S4). Additionally, *Planctomycetes* is the only persistent population to have at least one gene assigned to each COG category under positive selection.

### Nucleotide variation within core genomes

**Fig 5:**
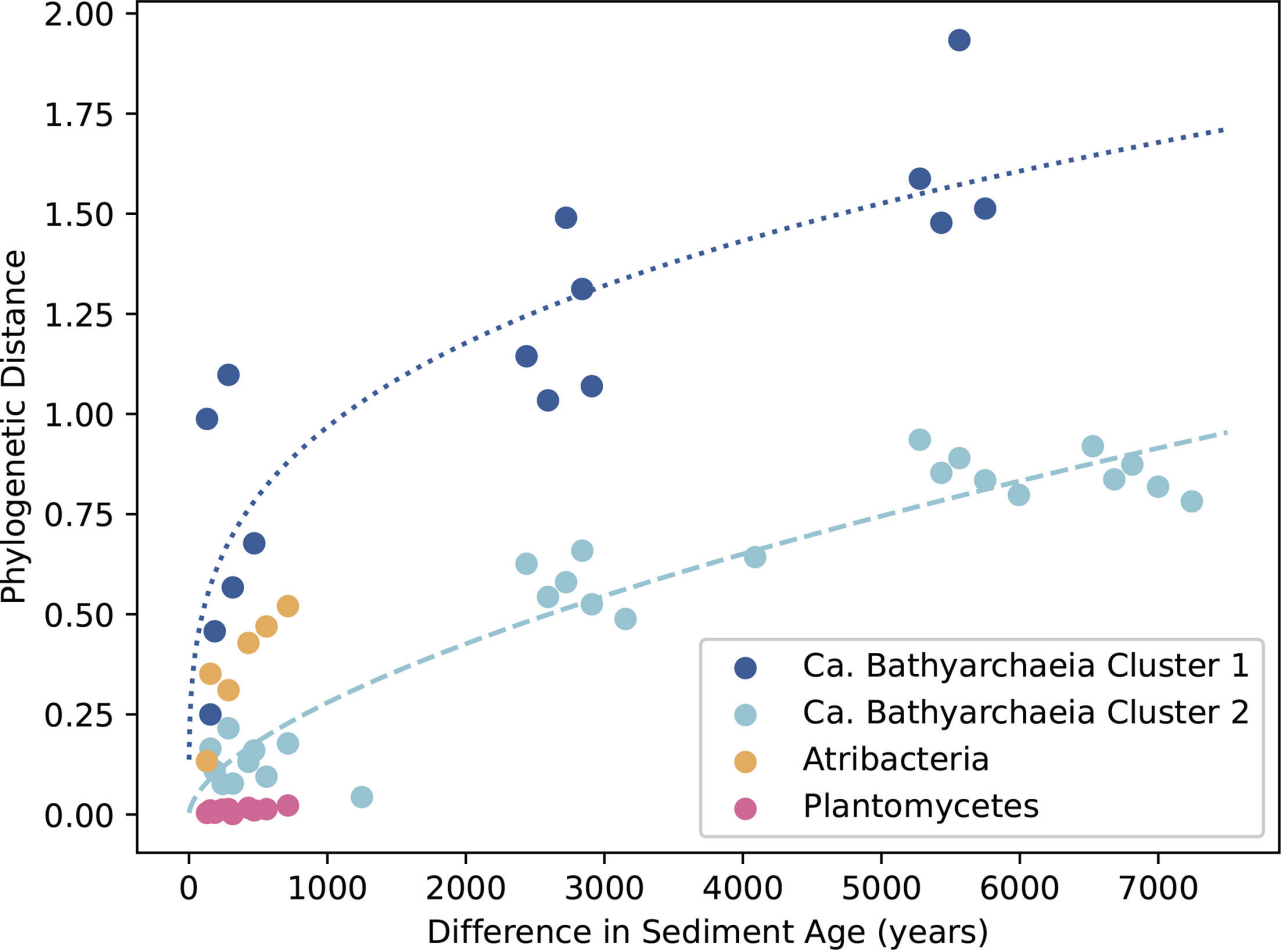
Correlation of phylogenetic distance and sediment age. The pairwise phylogenetic distance (y-axis) between two MAGs of a persistent population cluster (color) is plotted with respect to the difference in the age of the sediments from which the MAGs were retrieved (x-axis) and modeled by a power law ($D=aX^{b}$, dashed lines). *Ca*. Bathyarchaeia cluster 1 fit: $a=0.14,\;b=0.28,\;R^{2}=0.73$. *Ca*. Bathyarchaeia cluster 2 fit: $a=4.1\times 10^{-3},\;b=0.61,\;R^{2}=0.92$.

Phylogenetic analysis of each pangenome’s core genome indicates that there is a strong correlation between the phylogenetic distance of two MAGs and the difference in the sediment age from which the MAG was recovered with the largest phylogenetic distances observed within BC1 (Fig. 5). Here, the initially rapid increase in phylogenetic distance with difference in sediment age slows as sediment age differences exceed 2,000 years. By contrast, BC2 exhibits a phylogenetic distance versus sediment age pattern that can be explained by either a linear (*R*^2^ = 0.83) or power-law (*R*^2^ = 0.92) fit that is consistent with a neutral or weak selection model. Although undersampled relative to the other groups, *Planctomycetes* also exhibit a more linear increase in the pairwise phylogenetic distance with sediment age (Fig. 5). This clock-like (neutral) behavior across the sedimentary sequence is further reflected by the group’s extremely low SNP density and reduced proportion of genes under positive selective pressure (Table 2).

## DISCUSSION

Sedimentary prokaryotic communities in Lake Cadagno follow the global trends observed in marine and freshwater anoxic sediments (10). In these environments, a net decrease in microbial abundance and relative increase in the archaeal fraction is observed with depth, along with the cosmopolitan occurrence of persistent lineages (e.g., *Ca*. Bathyarchaeia, *Atribacteria*, *Dehalococcoidia* [phylum *Chloroflexi*], and *Planctomycetes*) (8, 11, 25–27). The persistence of these taxonomic groups in Lake Cadagno (Fig. 1) and the identification of related species (Table 3) across large geographic, salinity, and nutrient supply gradients are supportive of a long-standing association of these organisms with subsurface environments over geological timescales and are best exemplified by the BC1 lineage.

Despite the large differences in geographic distance, geographical context, and salinity of Lake Cadagno and Siberia, Russia’s Polar Fox Lagoon (28, 29), both sedimentary environments host BC1 populations with 98% ANI and lose only 21 core genes in the expansion of BC1’s local-to-global pangenome (Tables 2 and 3). Similar levels of genomic conservation across large geographic distances have also been observed in subsurface lineages such as the terrestrial subsurface-dwelling *Desulforudis audaxviator* (30, 31), cold seep sulfate-reducing bacteria SRB-SEEP1c (14), and cold seep anaerobic methane-oxidizing *Methanosarcina* (14); however, none of these pangenomic comparisons occur in environments with as large a salinity difference (1–2 orders of magnitude) as Lake Cadagno and Polar Fox Lagoon. It is worth noting, however, that the core genome of the local BC1 pangeome is already relatively small (38%) and a combination of biological and technical factors likely contributes to a dampened rate of core genome loss with the addition of new genomes to the pangenome (32). While we cannot exclude these factors, we also note that the core genomes of *Atribacteria* and *Dehalococcoidia* pangenomes are more affected by the addition of non-Lake Cadagno genomes, losing up to 55% of core genes through the addition of non-Lake Cadagno MAGs (Table 3).

In line with our observations of a sediment-adapted BC1 global pangenome, an evolutionary study of *Ca*. Bathyarchaeia proposes a geothermal sedimentary origin for the group and a conservation of adaptations toward sedimentary life (19). “Baizomonadales” (BC1’s taxonomic group) are the most widely distributed bathyarchaeial lineage on the planet, while members of “Wuzhiqibiales” (BC2’s taxonomic group) are more restricted in their distribution, being mainly found in anoxic sediments (19). In Lake Cadagno, BC2 replaces BC1 as the dominant *Ca*. Bathyarchaeia group at 738 cmblf (Fig. 1; Fig. S1). This transition is likely related to changes in carbon availability as the related BC1 and BC2 MAGs target different carbon pools (Fig. 4), and a significant decrease in TOC isotopic values (15) along with changes in carbohydrate compositional trends (16) has been observed throughout this region of the sedimentary sequence. More specifically, BC1 encodes the functional potential to break down carbohydrates, convert pyruvate to acetyl-CoA, and perform the bidirectional conversion of CO and CO_2_ through carbon monoxide dehydrogenase (*cdh*; Fig. S3) while also being significantly enriched in energy metabolism, electron transfer, and DNA replication and repair genes relative to BC2 (Table S2). On the other hand, BC2 MAGs are significantly enriched in protein-encoding genes related to peptide degradation (Table S3) and encode a suite of carbohydrate esterases (CE) related to peptidoglycan degradation and glycosyl hydrolases (GH) related to cellobiose degradation that BC1 does not (Fig. S3). These genomic predictions of the dominant *Ca*. Bathyarchaeia groups have been supported by ^13^C-labeled protein (33) and ^13^C-carbonate (34) incubation experiments that have demonstrated *Ca*. Bathyarchaeia’s utilization of extracellular proteins for peptide degradation and their ability to fix carbon in marine sediments. These local and global patterns of bathyarchaeial diversity may indicate that, with increasing sediment depth, “Wuzhiqibiales” will frequently replace “Baizomonadales” as the dominant bathyarchaeial group due to its ability to continually recycle peptides in sediments when bioavailable carbon sources become scarce, rather than gaining genes to target new carbon sources. This example of species replacement is consistent with environmental filtering for pre-adapted lineages, an ecological process frequently observed in other sedimentary environments (for a review, see reference 9).

Within this broader context of taxonomic turnover, previous studies on the microdiversity of sedimentary ecosystems have reported minimal intrapopulation genomic diversification over timescales ranging from thousands (6) to millions of years (35–37) after burial. Consistent with this, Lake Cadagno’s persistent populations exhibit even lower population-level diversity than the persistent lineages of a 5,000-year-old sedimentary sequence from Aarhus Bay (6), with core genomes characterized by low SNP densities and less than 10% of core genes under positive selection (Fig. 3A; Table 2), but maintain a signal of progressive isolation and divergence of populations with depth (Fig. 3B). However, because read-mapping-based metrics like those used in Fig. 3 primarily capture within-layer polymorphism and can underestimate divergence that becomes fixed among depths, we complemented them with phylogenetic and pangenomic approaches to examine genomic diversification with burial (Fig. 5). This analysis reveals a rapid accumulation of mutations over relatively short timescales (tens to hundreds of years) in BC1 and *Atribacteria*, followed by a slowdown in core genome changes as these populations decline in abundance over millennia along the sediment sequence (Fig. 5). This pattern may be driven by higher rates of population turnover in the organic carbon remineralization zone where an increase in total microbial abundance is also observed (10). By contrast, BC2 shows a slower but more constant accumulation of mutations in its core genome in the time since burial—consistent with a clock-like evolution under neutral or weak purifying selection—likely linked to its steadier and smaller effective population size and limited episodes of depth-specific adaptive change. Together, these patterns indicate that while overall microdiversity remains low, depth-structured diversification proceeds via early bursts of substitution in some lineages, whereas others accrue changes more uniformly through time.

The relatively large variable genomes enriched in genes related to energy conservation and transcriptional regulation at depth (Table S3) within the BC1 and BC2 pangenomes further point to selection during burial. For BC1, a decrease in genome size and pseudogene frequency with depth is observed (Fig. 2; Tables S1 and S2) and may indicate that genome streamlining is occurring within the BC1 lineage after burial. On the other hand, Lake Cadagno’s persistent *Atribacteria* and BC2 populations exhibit significantly greater population-wide pseudogene frequencies at 283 cmblf (Fig. 2) without evidence of genome streamlining at depth. For a collection of *Thalassospira* colonies cultivated from million-year-old abyssal clays, pseudogene abundance was correlated to sediment age and interpreted as the decay of accessory genes, like flagella, that were not needed for survival at depth (37); however, pseudogenes are also reported to rapidly accumulate in endosymbionts as they adapt to new hosts (38) and can be lost at different rates, depending on species. In the endosymbiont *Buchnera aphidicola*, the mean half-life of a pseudogene is estimated to be 23.9 million years (39), whereas pseudogenes are reported to be rapidly eliminated in *Salmonella* (40). While the lack of high-quality *Atribacteria* MAGs from depths greater than 283 cmblf prohibits us from investigating the fate of the pseudogenes that are uniquely encountered in this lineage at 283 cmblf (Table S2) and the mechanisms shaping the pseudogene patterns we observe in Lake Cadagno are currently unknown, the distinct patterns of pseudogene accumulation and loss within the BC1 and BC2 lineages, along with their differences in the number of core genes experiencing positive selection (Table 2) and phylogenetic divergence rates (Fig. 5) highlight lineage-specific evolutionary trajectories throughout the ~8,000 year Lake Cadagno sedimentary sequence.

Based on these findings, we conclude that persistent populations in Lake Cadagno sediments experience depth-associated selection during burial, albeit at lineage-specific rates. The initially dominant BC1 and *Atribacteria* show signs of adaptive evolution through the enrichment of energy conservation genes within their large variable genomes with depth and nearly 10% of their core genes under positive selection (Table 2). By contrast, BC2 exhibits a more clock-like tempo in core genome evolution and a much lower frequency of genes under positive selection (Table 2, Fig. 5). As the probability of a new mutation becoming fixed within a population is inversely related to population size (41), differences in BC1, BC2, and *Atribacteria* population sizes—namely the much larger BC1 and *Atribacteria* populations that dramatically decrease in abundance with depth and the more consistent but small within-sample populations of BC2—can help explain these patterns (Fig. 1; Fig. S1). When considered alongside depth-resolved patterns in pangenome composition, SNP structure, and pseudogene loads, we additionally observe that populations with expansive, plastic accessory gene pools (BC1, *Atribacteria*) show early depth-associated adaptive change that tapers as populations decline and drift gains influence, whereas the more consistently small BC2 population accumulates substitutions at a steadier, clock-like pace under stronger purifying constraints. While these patterns are striking, they also come with important caveats. The number of MAGs per lineage is modest, all MAGs are fragmented rather than closed, and contamination from binning may artificially inflate the size of each population’s variable genome. We, therefore, recommend that future work targets single-cell and closed genome analyses to mitigate potential technical artifacts. Nevertheless, the depth-associated genomic changes observed across lineages in this study are striking and suggest that Lake Cadagno’s persistent populations follow distinct eco-evolutionary trajectories as they become progressively isolated through burial, encouraging future investigations of this topic in other advection-limited, anoxic sedimentary ecosystems and over even longer geological timescales.

## MATERIALS AND METHODS

### Sample collection and DNA sequencing

Lake Cadagno is a sulfidic, meromictic lake located in the Piora Valley in Switzerland. Since its formation ~12,000 years ago (17), the water column of the lake underwent redox transitions in the water column from oxic to episodically anoxic, to meromictic and euxinic (15, 17, 42). In August 2019, a 10 m sediment core was retrieved, and a detailed description of the coring, sampling strategy, and geochemistry can be found in reference 15. The uppermost lithological unit, which corresponds to iron-sulfide-rich sediments deposited in the Holocene, contains layers of laminated sediment from pelagic origin and mass-movement deposits with contributions from lacustrine and terrestrial origin. This study includes data from nine depths selected using lithostratigraphic and geochemical criteria (10) to capture environmental variability in the sedimentary sequence (Table 4) and represent ~8,000 years of sedimentary history based on age-depth modeling using ^14^C radiocarbon dating (15, 17). Further details on the sampling campaign and sample storage conditions can be found in reference 15.

**TABLE 4:** General information for the nine samples analyzed in this study^*a*^

Sample depth (cmblf)	Sediment age (yr BP)	Sediment type
40	221	Turbidite
153	1,026	Turbidite
187	1,270	Turbidite
213	1,456	Pelagic
233	1,586	Turbidite
283	1,741	Pelagic
582	3,472	Turbidite
693	7,019	Turbidite
738	8,268	Turbidite

^*a*^ The sediment ages are listed in years before present (yr BP) and were estimated using a model based on ^14^C calibrated ages (15). The sediment types correspond to the different lithologies of the sedimentary sequence. A more detailed geochemical description of these samples can be found in reference 10.

DNA was extracted using a modular protocol (for details, see reference 43), optimized to increase yield from old sediments. Further description of the extraction protocol can be found in reference 10. 16S rRNA gene amplicon sequencing and quantification using the universal primers 515F (5′-GTG YCA GCM GCC GCG GTA A-3′) and 926R (5′-CCG YCA ATT YMT TTR AGT TT-3′), targeting the V4–V5 region was performed and further described in reference 10. Shotgun metagenomic libraries were prepared using the NEBNext Ultra II DNA kit with sonication-based fragmentation and sequenced on an Illumina NovaSeq 6000. Oxford Nanopore sequencing was performed using the SQK-NBD114.24 kit on a PromethION 2 Solo with FLO-PRO114M (R10.4.1) flow cells, following a modified ligation protocol (44). Briefly, AMPure XP bead cleanup incubation times were extended from 5 to 10 min. To enrich DNA fragments ≥3 kb, Long Fragment Buffer was used during the first cleanup step following adapter ligation. For the second cleanup, Short Fragment Buffer was applied to retain fragments of all sizes. Base calling and demultiplexing were performed using Guppy v.6.5.6 (45) with super high accuracy.

### Illumina shotgun metagenomic data assembly and binning

The Illumina sequencing data were processed as described in reference 10. Briefly, sequences were quality-filtered and trimmed with Trimmomatic v0.35 (46) and assembled with SPAdes v3.14.1 (metaSPAdes.py) (47). Contigs over 1,000 bp were kept for further analysis. Each sample was binned individually using MetaWRAP (48). MAGs meeting the MIMAG (49) thresholds of ≥90% completeness and ≤5% contamination based on CheckM (18) scores were selected for downstream analyses.

### Selection of persistent populations for MAG analysis

A set of persistent groups was identified based on the concept introduced by reference 6, which defines them as members of the community that are consistently present throughout a sediment column. Here, persistent groups are defined as 16S rRNA gene amplicon sequencing variants (ASVs) present in ≥80% of the samples (10). Based on this definition, ASVs from *Chloroflexi*, *Ca.* Bathyarchaeia, *Planctomycetes*, and *Atribacteria* were identified as persistent groups. In the present study, we focused on these groups for MAG analysis. Persistent populations were identified as high-quality MAGs clustered at 98% ANI using dRep (22). As a result, clusters of MAGs at >98.5% ANI, referred to here as “populations,” were identified. Each cluster included one representative MAG from three or more distinct sample depths. With these criteria, no depth contained multiple MAGs belonging to the same population, meaning that within a given cluster, a single unique MAG was recovered per depth.

### Hybrid binning using Illumina and Oxford Nanopore sequencing data

Illumina MAGs were combined with the Oxford Nanopore Technology (ONT) sequencing data to improve contiguity and produce high-quality hybrids. In brief, Oxford Nanopore reads were mapped to Illumina-derived MAGs using Minimap2 (50) with the map-ont preset and parameters:-A 4 -O 6,30 -E 3,2. The resulting SAM files were converted to sorted and indexed BAM files using Samtools v1.16.1 (51). Mapped reads were extracted in FASTQ format using the samtools fastq command with the -F 4 flag, which retains only the reads aligned to the reference.

Hybrid assemblies were performed using SPAdes v3.15.5 (52) with the careful option to reduce mismatches and short indels during assembly. This mode also runs the MismatchCorrector tool, which utilizes BWA (21) for post-assembly refinement. Two separate hybrid assemblies were performed for each MAG: one incorporating strict short-read FASTQ files and the other permissive short-read FASTQ files, both paired with ONT long reads to improve contig contiguity. Contigs in the hybrid assemblies that were shorter than 5,000 bp were excluded from downstream analyses. The resulting hybrid assemblies from the strict and permissive short-read mapping strategies were processed separately for binning using the MetaWRAP (48) initial binning and bin refinement modules.

Illumina reads were mapped to hybrid bins using BWA (21). These alignments, poly-N content, and kmer content of bins were used to manually curate the hybrid bins. Each MAG produced from these strategies was evaluated for quality using CheckM v1.0 (18) to calculate quality statistics (see Table S1). Based on the results, the best MAG from either the strict or permissive assembly was selected as the representative MAG for each sediment sample depth within the 98.5% identity MAG clusters or “populations.” Functional and taxonomic annotation was performed on the resulting hybrid MAGs using DRAM (53) and the GTDB-Toolkit (20), respectively. The relative abundance of each MAG per sample was assessed using CoverM (54) and the estimated qPCR abundances of each MAG (Fig. 1) were made using these relative abundance values and the qPCR results from reference 10.

### Pangenome construction and functional annotation

Pangenome construction for the persistent population clusters was performed using PPanGGOLiN v2.2.1 (55) using the workflow module. Pangenome partitions were manually defined based on the output files using the following criteria: the core genome comprises gene families present in all MAGs and represents the set of predicted gene families. The shell genome comprises gene families present in any integer number *n* of MAGs between 2 and *N* − 1 (inclusive), where *N* is the total number of MAGs in the cluster. The cloud genome consists of gene families unique to each MAG. Functional annotation of the predicted protein families from the PPanGGOLiN output was performed using eggNOG-mapper v2.1.6 with a likelihood threshold of 1 × 10^−5^. Pseudogenes were annotated using DFAST (56), and the relative abundance of each MAG was computed using CoverM (54).

A local pangenome was generated for each persistent group from Lake Cadagno. Note that the *Dehalococcoidia* MAGs were analyzed alongside their closest GTDB relatives and the *Atribacteria* population was analyzed with a 97.5% ANI relative from 40 cmblf to meet PPanGGOLiN’s minimum requirement of five genomes for pangenome construction. Global pangenomes were also made for BC1 and *Atribacteria* by adding their closest GTDB relatives to the genome set analyzed by PPanGGOLiN.

### Phylogenetic analyses

Multiple sequence alignments of each local pangenome’s core genome were obtained via the PPanGGOLiN msa --phylo module. SNPs within each core genome’s multiple sequence alignment were quantified using SNP-sites (57). A phylogenetic tree from each core genome’s multiple sequence alignment was generated using RAxML and the GTR+R model (58). The resulting best tree from 100 bootstrap trees was used in association with the codeml algorithm of PAML to perform the *dN*/*dS* analysis on the genes within each pangenome’s core genome (24). Microdiversity analyses were performed by mapping each metagenome onto each population’s representative MAG using BWA. These alignments were then used as inputs to inStrain for microdiversity analyses. Single-cell amplified genomes and metagenomes from Aarhus Bay (6) were additionally retrieved from their respective public databases for the microdiversity analyses described above, with metagenomic reads mapped to each single-cell genome. A minimum inStrain breadth of 0.8 and coverage of 10 were used as thresholds for subsequent microdiversity analyses. *F*_ST_ was calculated using the SNP calls from inStrain and the definition of reference 23.

## Data Availability

The shotgun metagenomic sequencing data have been deposited in the NCBI database under BioPproject number PRJNA1160237. The Oxford Nanopore sequencing data have been deposited in the NCBI database under BioPproject PRJNA1430377. The metagenome-assembled genome data set used to produce the figures presented in this study can be found at https://github.com/GeobiologyLab/Rodriguez_etal_mSystems2026.

## SUPPLEMENTAL MATERIAL

Data S1 msystems.01500-25-s0001.xlsx Persistent MAG annotation files.

Supplemental figures msystems.01500-25-s0002.pdf Figures S1 to S3.

Supplemental legends msystems.01500-25-s0003.txt Legends for supplemental material.

Table S1 msystems.01500-25-s0004.xlsx Extended table of MAG statistics.

Table S2 msystems.01500-25-s0005.csv Pseudogene statistics.

Table S3 msystems.01500-25-s0006.xlsx Pangenome enrichment test results.

Table S4 msystems.01500-25-s0007.xlsx Core genes under positive selection.

ASM does not own the copyrights to Supplemental Material that may be linked to, or accessed through, an article. The authors have granted ASM a non-exclusive, world-wide license to publish the Supplemental Material files. Please contact the corresponding author directly for reuse.
