# Supplementary material for: Depth-associated selection and drift shape persistent microbial populations in Holocene lake sediments
Source: mSystems. 2026 May 5;11(6):e01500-25. doi: 10.1128/msystems.01500-25 (PMC13288992; doi:10.1128/msystems.01500-25)
Supplement: Supplemental figures — Figures S1 to S3. [file msystems.01500-25-s0002.pdf]

Supplementary Figure 1.

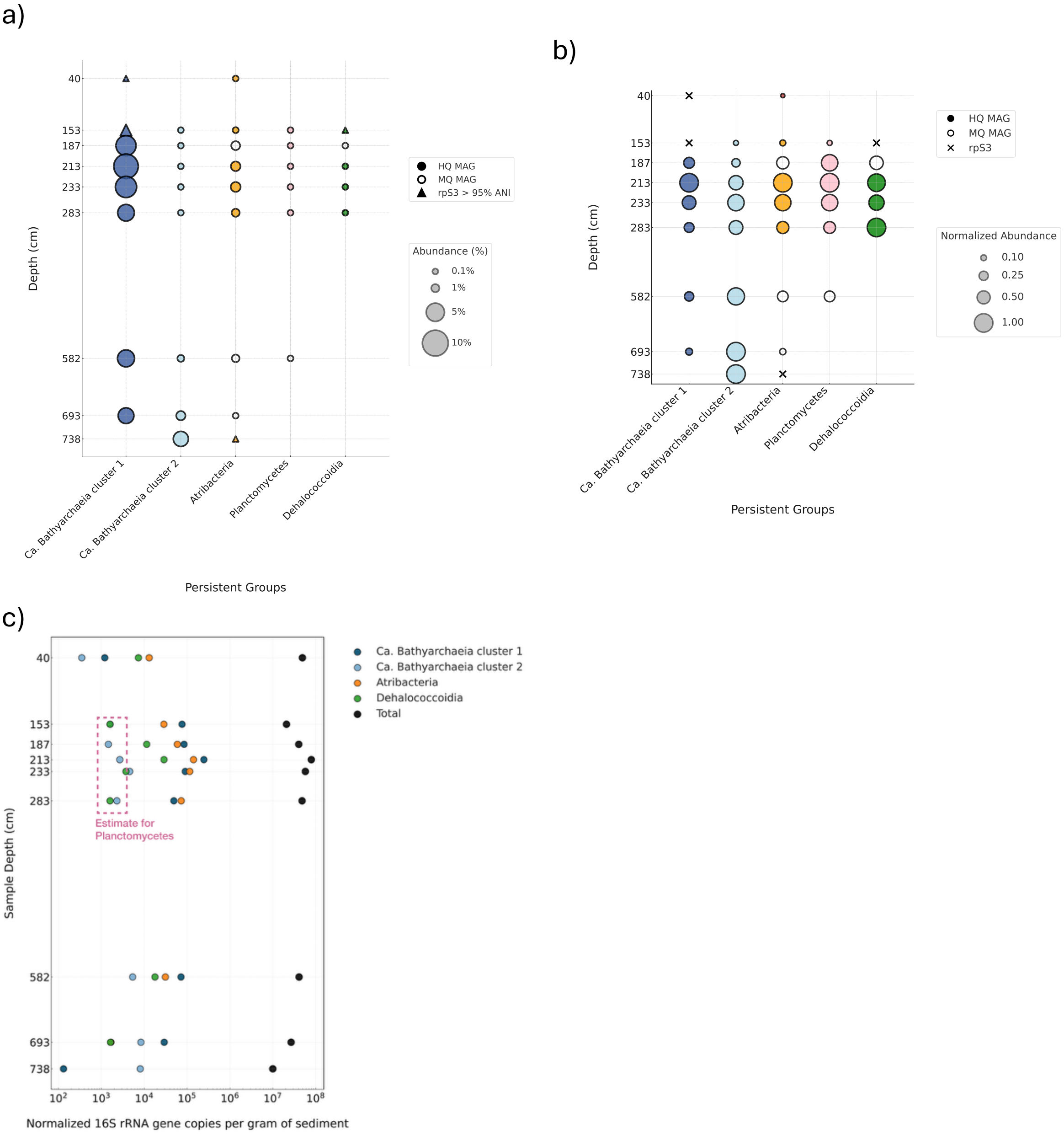

(a) The relative abundance (dot size) of persistent lineages (x-axis) with depth (y-axis) is shown. Medium quality (MQ) MAGs (open circles) and *rpsS3* gene sequences (triangles) with ≥98% average nucleotide identity (ANI) to high quality (HQ) MAGs were added for completeness. (b) The depth-specific relative abundance of a population cluster normalized against its maximal relative abundance (dot size) is shown. The red HQ *Atribacteria* MAG at 40 cm depth indicates a 97.5% ANI relative of the *Atribacteria* persistent population. (c) Total normalized 16S rRNA gene abundance (x-axis) for the persistent groups in Lake Cadagno (see legend). The values represent the relative abundance of 16S rRNA amplicon sequencing variants (ASVs) associated with the MAGs from the persistent groups, normalized to 16S rRNA gene copies per gram of sediment. ASVs related to the persistent *Planctomycetes* MAG 16S rRNA gene were not identified in the 16S rRNA gene amplicon dataset and, therefore, the abundance of this group was estimated according to the metagenome-derived relative abundance patterns of (a).

Supplementary Figure 2.

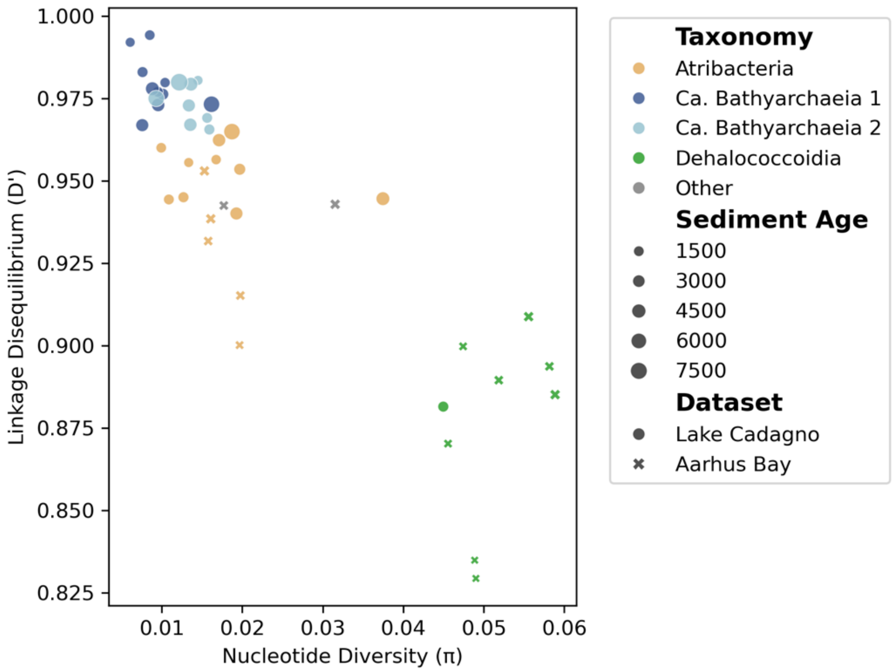

The inStrain computed D' linkage disequilibrium metric (y-axis) relative to nucleotide diversity (π, x-axis) for MAGs from Lake Cadagno (circles) and Aarhus Bay (crosses) are shown. A lower D' is indicative of higher levels of homologous recombination.

Supplementary Figure 3.

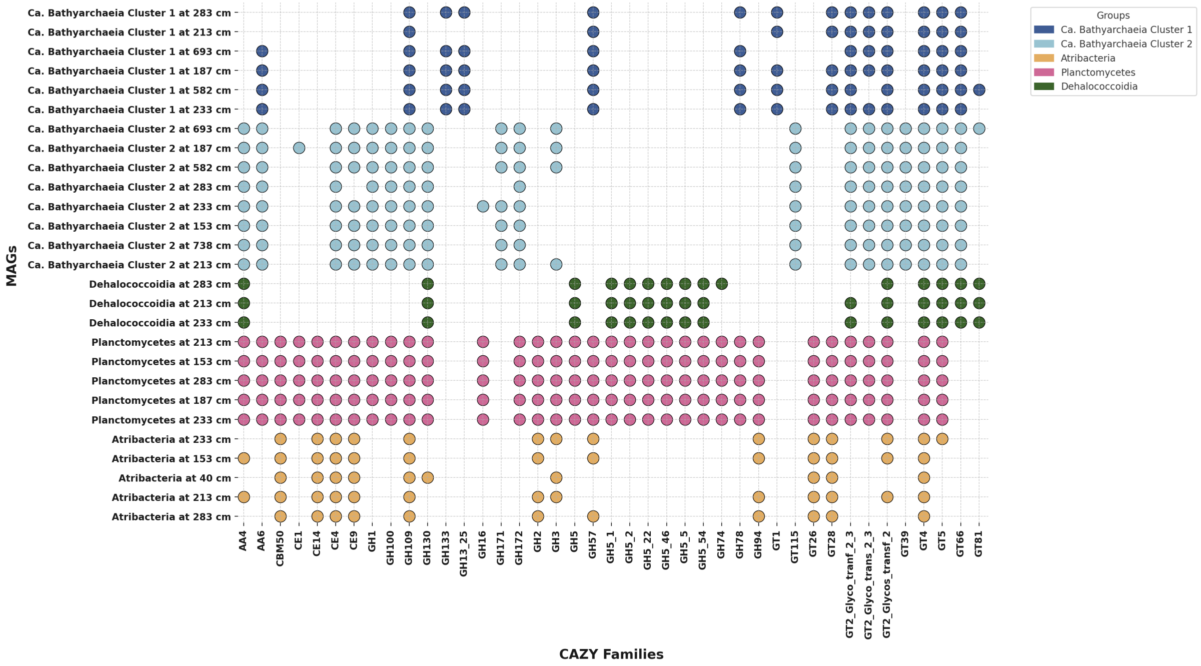

Distribution of CAZy families across MAGs assigned to persistent population clusters. Each point indicates the presence of a CAZy family in a MAG. With the exception of *Ca.* Bathyarchaeia MAG CAZy families, only CAZy families appearing in at least 2 persistent lineages are shown. CAZy families are classified as Glycoside Hydrolases (GH), Glycosyltransferases (GT), Carbohydrate Esterases (CE), Polysaccharide Lyases (PL), Auxiliary Activities (AA), and Carbohydrate-Binding Modules (CBM).
